# Structural interplay of anesthetics and paralytics on muscle nicotinic receptors

**DOI:** 10.1038/s41467-023-38827-5

**Published:** 2023-06-01

**Authors:** Umang Goswami, Md Mahfuzur Rahman, Jinfeng Teng, Ryan E. Hibbs

**Affiliations:** 1 Department of Neuroscience and O’Donnell Brain Institute, University of Texas Southwestern Medical Center, Dallas, TX 75390 USA; 2 Department of Neurobiology, University of California, San Diego, La Jolla, CA 92093 USA; 3 Present Address: Thermo Fisher Scientific, Rockford, IL 61101 USA

**Keywords:** Ion channels in the nervous system, Molecular medicine, Permeation and transport, Cryoelectron microscopy, Ligand-gated ion channels

## Abstract

General anesthetics and neuromuscular blockers are used together during surgery to stabilize patients in an unconscious state. Anesthetics act mainly by potentiating inhibitory ion channels and inhibiting excitatory ion channels, with the net effect of dampening nervous system excitability. Neuromuscular blockers act by antagonizing nicotinic acetylcholine receptors at the motor endplate; these excitatory ligand-gated ion channels are also inhibited by general anesthetics. The mechanisms by which anesthetics and neuromuscular blockers inhibit nicotinic receptors are poorly understood but underlie safe and effective surgeries. Here we took a direct structural approach to define how a commonly used anesthetic and two neuromuscular blockers act on a muscle-type nicotinic receptor. We discover that the intravenous anesthetic etomidate binds at an intrasubunit site in the transmembrane domain and stabilizes a non-conducting, desensitized-like state of the channel. The depolarizing neuromuscular blocker succinylcholine also stabilizes a desensitized channel but does so through binding to the classical neurotransmitter site. Rocuronium binds in this same neurotransmitter site but locks the receptor in a resting, non-conducting state. Together, this study reveals a structural mechanism for how general anesthetics work on excitatory nicotinic receptors and further rationalizes clinical observations in how general anesthetics and neuromuscular blockers interact.

## Abstract (web summary)

Here the authors use a structural approach to reveal how neuromuscular blockers and a general anesthetic antagonize the muscle-type nicotinic receptor through competitive and allosteric mechanisms.

## Introduction

Nicotinic acetylcholine receptors on muscle fibers are inhibited by many general anesthetics and are the principal targets of neuromuscular blockers used in surgery^1^. General anesthetics act mainly by targeting ligand-gated ion channels in the nervous system including those of the Cys-loop receptor family^2,3^. Within this pentameric channel family, intravenous anesthetics such as etomidate potentiate inhibitory GABA_A_ receptors and inhibit excitatory nicotinic acetylcholine receptors^4,5^. These combined actions lead to an overall reduction of neuronal activity^5,6^. Neuromuscular blockers, or paralytic agents, antagonize the muscle nicotinic acetylcholine receptor thereby disrupting signaling between motor neurons and skeletal muscle. They are used in combination with general anesthetics to enable muscle relaxation and to lower the amount of general anesthetic required during surgery, which improves safety and hastens recovery^7^. General anesthetics themselves also inhibit the muscle-type nicotinic receptor^4,8–11^; isoflurane, for example, is known to increase the efficacy of the neuromuscular blocker succinylcholine^12^. How general anesthetics modulate the muscle-type nicotinic receptor and alter the activity of neuromuscular blockers remains poorly understood.

Neuromuscular blocking agents fall into two categories. Depolarizing blockers cause transient nicotinic receptor channel opening and muscle fasciculations before stabilizing a nonconducting, desensitized channel state. This prolonged desensitization results in skeletal muscle paralysis. Succinylcholine is the most commonly used drug in this category^13,14^. Non-depolarizing blockers like rocuronium do not cause channel opening but instead stabilize a distinct nonconducting channel state resulting in paralysis. Both muscle relaxants directly compete with acetylcholine and thereby block its action at the muscle nicotinic receptor, and both are used for rapid onset of muscle paralysis and aid in endotracheal intubation. Direct structural insights into how these clinically useful neuromuscular blockers bind to and antagonize the muscle-type nicotinic receptor are absent.

Here we used cryo-electron microscopy (cryo-EM) and electrophysiology to understand how general anesthetics and neuromuscular blockers act at the muscle-type nicotinic receptor, and to provide a foundation for understanding drug interactions. We report four high-resolution cryo-EM structures of a muscle-type nicotinic receptor in complex with the general anesthetic etomidate and the agonist choline, with the depolarizing blocker succinylcholine, and in two distinct states bound to the non-depolarizing blocker rocuronium. We first define where etomidate binds and how its binding affects receptor conformation and activity. We then contrast the anesthetic and blocker bound structures of the receptor to reveal common and distinct mechanisms of inhibition. We use electrophysiology to relate observed structures to physiological states. Together, we discover a negative modulator binding site for etomidate and elucidate inhibition mechanisms potentially relevant to drug interactions in clinical anesthesia.

## Results

### Biochemistry and receptor architecture

**Fig. 1:**
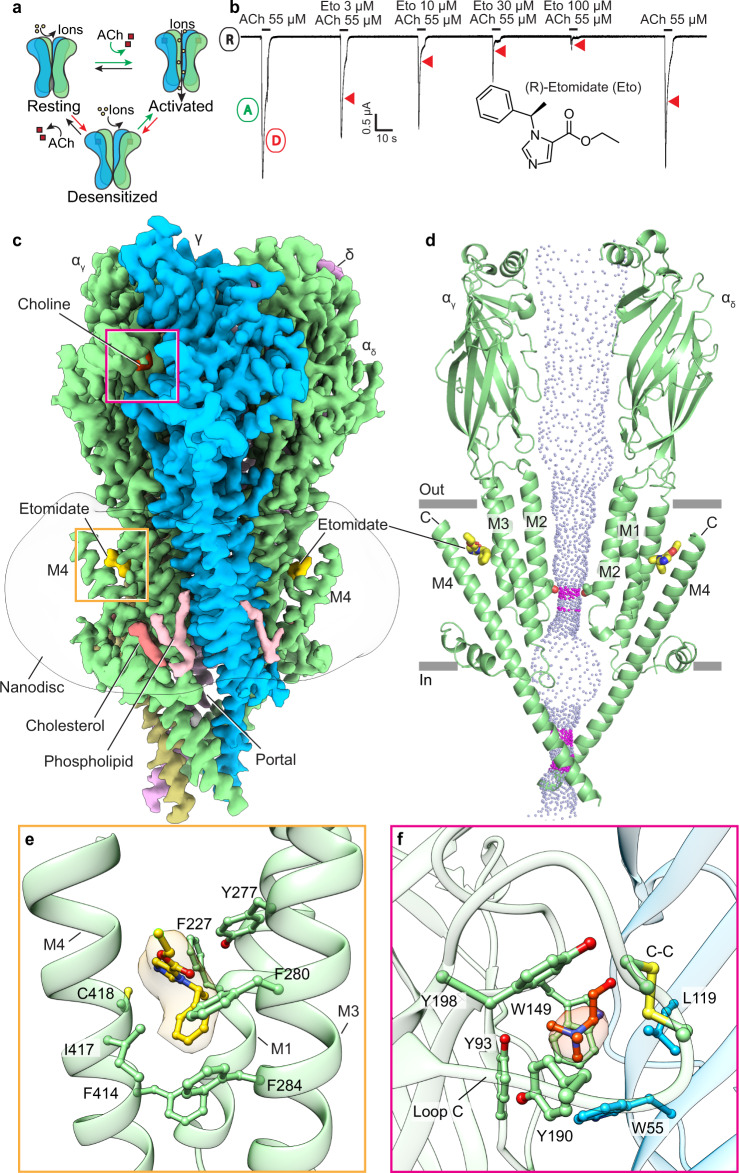
Etomidate stabilizes a desensitized state through TMD binding. **a** Simplified gating cycle cartoon for the nicotinic receptor. Acetylcholine (ACh) binding shifts the conformational equilibrium toward a conducting, activated state, then to the more stable nonconducting desensitized state. **b** Two-electrode voltage clamp (TEVC) electrophysiology shows activation by acetylcholine and dose-dependent receptor antagonism by etomidate (Eto). R, A, and D indicate channel resting state, and activation and presumed desensitization components of current response. Red triangles highlight etomidate increasing apparent desensitization rate. Incomplete recovery in final acetylcholine application is from slow washout of etomidate, consistent with its membrane partitioning. Similar responses were seen from *n* = 8 independent cells. **c** Side view of the overall cryo-EM map of the receptor-etomidate-choline complex; α subunit is in green, β subunit in khaki, γ subunit in blue, δ subunit in violet, choline in red, etomidate in gold, cholesterols in tomato red, and phospholipids in pink; the lipid nanodisc is shown as a semitransparent surface. α_γ_ and α_δ_ subunits are named based on their neighboring complementary subunits. **d** Side view of two α subunits showing permeation pathway as dots representing solvent-accessible surface colored by diameter; purple is 2.8–5.6 Å diameter, while diameter > 5.6 Å is shown in blue. Thr2ʹ that forms the pore constriction is shown as sticks. **e** Etomidate-receptor interactions in α_γ_ subunit are shown as sticks with corresponding density. **f** Choline at α/γ interface.

We purified the muscle-type nicotinic acetylcholine receptor from the electric organ of *Torpedo californica* and reconstituted it into lipid nanodiscs for structure determination by cryo-EM^15^. This receptor has long served as a model system for the human muscle receptor^16–20^, and retains the relevant pharmacology for neuromuscular blockers and etomidate^9,21–23^. The receptor assembles as a pentamer from four homologous subunits arranged α-γ–α–δ–β pseudo-symmetrically around the channel axis (Fig. 1 and Supplementary Fig. 1)^24–26^. The large extracellular domain (ECD) harbors the classical neurotransmitter binding sites at α–γ and α–δ interfaces^24^. The transmembrane domain (TMD) of four α-helices per subunit forms a pore lined by the M2 helices. The intracellular domain (ICD), which is partly disordered in all known structures, comprises extensions of the M4 helix that frame lateral portals for tuning ion conductance. We preformed complexes of the receptor with etomidate, succinylcholine, and rocuronium before freezing samples for electron microscopy. This approach enabled determination of structures to overall resolutions of 2.7–2.9 Å, with density quality sufficient for modeling ligands and all of the receptor ECD, TMD, and all of the predicted intracellular MX and MA helices (Fig. 1, Supplementary Table 1, Supplementary Figs. 1 and 2, Supplementary Movies 1–9).

### Etomidate acts through an intrasubunit TMD site

The general anesthetic etomidate inhibits the activity of the muscle-type nicotinic receptor at concentrations that occur during clinical anesthesia^7,10,11,27^. Photoaffinity labeling studies have suggested several potential etomidate binding sites^23,27–30^. We performed electrophysiology experiments to better understand which steps in the channel gating cycle etomidate stabilizes: resting, activated, or desensitized (Fig. 1a, b). We observed that etomidate reduced the peak agonist response and further found that etomidate increased the current decay rate upon agonist exposure (Fig. 1b). This increase in decay rate likely stems from enhancing desensitization at low concentrations coupled with channel block at higher etomidate concentrations as suggested by photoaffinity studies^27,29^. Together these results indicate that etomidate’s inhibitory activity at concentrations used during anesthesia (EC_50_ for anesthesia of 2–4 μM^7,10,28^) may stem from stabilizing a nonconducting desensitized state, to which agonists bind most tightly. Notably, photoaffinity based studies found that during brief activation, etomidate principally acts by inhibiting the activated state^23^, while at an equilibrium accessible more readily by structural biology, etomidate stabilizes a desensitized channel state^27^. To provide a structural framework for where etomidate binds, and how it inhibits the channel, we obtained a cryo-EM structure of the muscle-type nicotinic receptor bound to etomidate at 2.8 Å overall resolution (Fig. 1c–e, Supplementary Figs. 1 and 2, Supplementary Table 1).

The cryo-EM map revealed unambiguous density for etomidate within the transmembrane domain of each principal (α) subunit (Fig. 1c–e). This intrasubunit site came as a surprise as it is strikingly distinct from etomidate’s site in the homologous GABA_A_ receptor. In this related anion-selective channel, etomidate binds at β-α subunit interfaces and acts as a positive modulator (Fig. 1c, Supplementary Fig. 3)^31^. The muscle receptor binding site, in contrast, is formed by the extracellular ends of the α-subunit M1, M3, and M4 helices. The accessibility of this site depends upon detachment of the M4 C-terminus from the receptor, which is observed at least partially in all agonist-bound structures of the muscle-type nicotinic receptor (Fig. 1c–e and Supplementary Fig. 4). Indeed, in previous work we determined that conformational transitions in this site contributed to recovery from desensitization^20^. The pose of etomidate is equivalent at both its α-subunit binding sites, with its phenyl ring orienting toward the intracellular side of the membrane, the imidazole ring normal to the membrane and the ester group pointing toward the extracellular side (Fig. 1e). The phenyl ring of etomidate makes π-stacking and Van der Waals interactions with surrounding phenylalanine residues (M4-F414, M3-F284 and M3-F280). Etomidate further forms hydrophobic contacts with M4-I417, M4-C418, M1-F227 and M3-Y277. These amino acids are conserved among *Torpedo*, mouse, and human receptors. Only M4-I417 is substituted by valine in humans. The binding site conservation suggests that observations for etomidate effects through this site would translate to mammalian species including humans. Mutagenesis of binding site residues did not reveal large changes in etomidate inhibition, perhaps due to the flexible nature of the binding site and the lack of electrostatic interactions made by the drug (Supplementary Fig. 5). The α-M4 helix was identified previously from photolabeling studies^27^ to be a potential etomidate binding site (Supplementary Fig 6). However, the labeled residues in M4 do not orient toward the observed drug binding pocket. Mismatches between results from electron microscopy and photolabeling may stem from the latter being able to highlight lower occupancy, lower affinity sites, and from the photoreactive etomidate derivatives binding differently from the unmodified compound. Additional density specific to the etomidate-bound structure was apparent but not modeled in the upper half of the pore and extending from M4-M415 and M4-C418 (Supplementary Fig. 7 and Supplementary Movie 10). The pore density, which was diffuse, is consistent with a low affinity pore block site for etomidate suggested by photoaffinity studies^27^. The density extending from the M4 cysteine, adjacent to bound etomidate, may arise from a lipid that becomes ordered when etomidate is bound, or from the *N*-ethylmaleimide treatment used to prevent disulfide crosslinking during purification.

We obtained the etomidate-bound receptor structure using a purification strategy previously optimized to determine the apo, resting state of the receptor^20^. However, the etomidate complex cryo-EM map revealed density consistent with the weak agonist choline positioned in the classical neurotransmitter binding pocket (Fig. 1f). A very high concentration of choline (1.2 M) was used to elute the receptor during affinity chromatography but was then omitted from subsequent size exclusion chromatography and is not seen in our apo receptor map. The structural results suggest that etomidate increases the affinity for agonists in the neurotransmitter binding pocket, observed in binding measurements^27^, so much so that choline stays bound to the receptor throughout the final steps of purification. Consistent with bound agonist, loop C packs down tightly on the subunit interface, stabilizing the positively charged ammonium in an aromatic box (Fig. 1f) defined in earlier structures^24,32^. This compaction of loop C is part of a structural rotation and compaction of the ECD as a whole seen across the receptor superfamily^33^. Agonists are known to bind most tightly, at equilibrium, to the desensitized state; that etomidate stabilizes agonist binding is in line with its inhibitory activity being connected to enhancing desensitization.

The structure of the muscle-type receptor bound to etomidate and choline reveals a permeation pathway consistent with a desensitized state (Fig. 1d). The extracellular vestibule is widely open, and the transmembrane pore, comprising M2 helices from each of the five subunits, tapers from relatively wide on its extracellular end to a constriction point near the junction with the cytosol. The minimal pore diameter is 4.5 Å and is defined by threonine side chains at the 2ʹ position, similar to what was observed in complexes with the agonists carbachol and nicotine^19,20^. While polar constrictions of this diameter are suggested to permit some ion passage in simulations^19^, this diameter is much smaller than the 7.4 Å minimum diameter estimated from ion permeability studies for an activated state^34^. Together, the functional and structural analyses support etomidate acting through a formerly uncharacterized intrasubunit TMD site to stabilize a nonconducting, desensitized-like channel state.

### Depolarizing blocker succinylcholine stabilizes a desensitized state

**Fig. 2:**
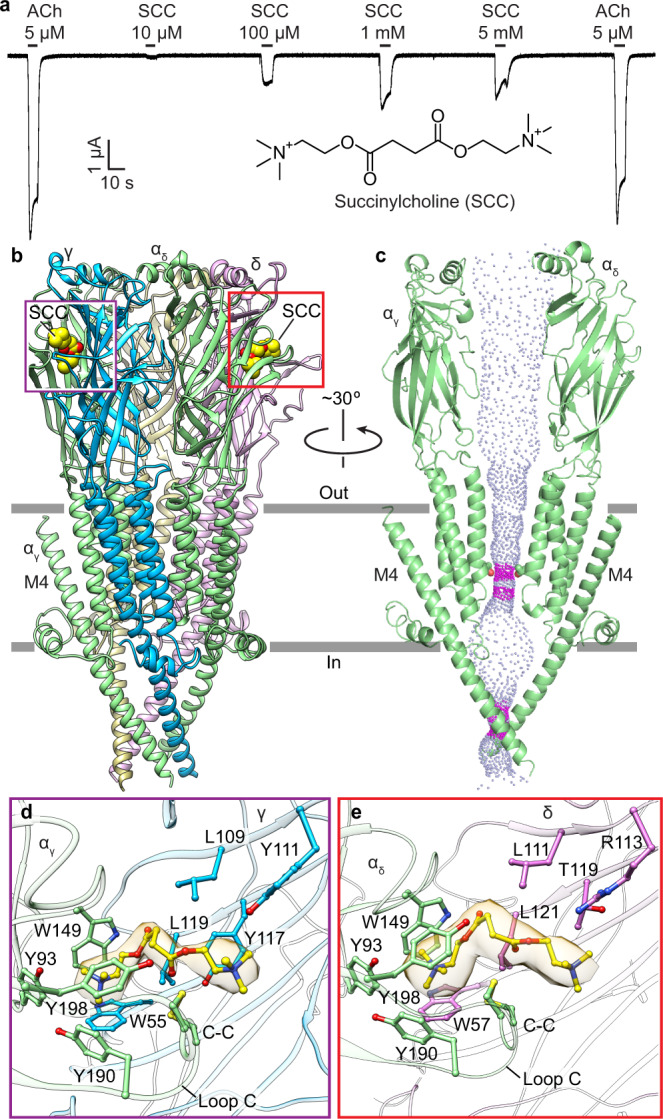
Succinylcholine inhibition and binding mechanism. **a** TEVC electrophysiology illustrates lower efficacy of succinylcholine (SCC) compared to acetylcholine (ACh). Similar responses were seen in *n* = 8 independent cells. **b** Side view of succinylcholine-bound structure. Subunits are colored as in Fig. 1. Succinylcholine is shown as yellow spheres. **c** Side view of two α subunits showing permeation pathway as dots representing solvent-accessible surface colored by diameter; Thr2ʹ that forms pore constriction is shown as sticks. **d** Succinylcholine at α/γ interface. **e** Succinylcholine at α/δ interface.

Succinylcholine is the most common neuromuscular blocker used during general anesthesia. This paralytic agent comprises two linearly linked copies of acetylcholine, with a quaternary ammonium pharmacophore on each end (Fig. 2a). It is an orthosteric agonist for the *Torpedo* nicotinic receptor and the human muscle nicotinic receptor but not the neuronal nicotinic receptors^35,36^. The paralytic activity of succinylcholine stems from its resistance to hydrolysis by acetylcholinesterase, which extends its lifetime in the synapse compared to the neurotransmitter, allowing it to briefly activate, then stably desensitize the receptors. Electrophysiology experiments further illustrate its low efficacy compared to acetylcholine and reveal rebound currents upon washout at high concentrations, indicative of channel block (Fig. 2a). To address questions of specificity and mechanism of action of this important anesthetic adjunct, we obtained a cryo-EM structure of the *Torpedo* nicotinic receptor in complex with succinylcholine at an overall resolution of 2.7 Å. The map quality revealed clear density for succinylcholine bound in the classical neurotransmitter sites at extracellular α–γ and α–δ subunit interfaces (Fig. 2b, Supplementary Fig. 8a–c). Diffuse pore density in this structure is also consistent with the low affinity channel blocking activity (Supplementary Fig. 7).

Succinylcholine binds in a similar pose in both neurotransmitter sites, with one of its ammonium groups anchored in the classical aromatic box, while its linker and second ammonium snake away from the membrane and toward the complementary subunit. Interactions with the γ and δ subunits go beyond those seen with choline and other smaller agonists and are likely responsible for the subtype selectivity of this drug versus the neuronal receptor subtypes^36^. Comparison of the two binding sites within the muscle-type receptor reveals a lack of conservation in complementary subunit interactions, where γY111 and γY117, which interact with the second ammonium, are replaced with δR113 and δT119 (Fig. 2d, e, Supplementary Fig. 8b, c). Among these, δT119/γY117 stands out. In the neuronal complementary subunit β2, the δT119/γY117 position is a phenylalanine (β2F119). The substitution of threonine/tyrosine with a more hydrophobic phenylalanine may disrupt the interaction of succinylcholine with a neuronal β2 subunit. The neuronal β4 subunit has a leucine (β4L121) at the δT119/γY117 position; this very hydrophobic side chain again may disfavor interactions with succinylcholine. When the neuronal α7 subunit forms the complementary side of the binding pocket, the δT119/γY117 position in α7 is a glutamine (α7Q116). Superposition of the *Torpedo* receptor-succinylcholine complex with the α7 structures suggests that this glutamine would clash with the bound succinylcholine molecule and would thereby disfavor high-affinity succinylcholine binding. Furthermore, the cryo-EM map suggests that two loop C conformations are present in the α_γ_ subunit: one packed down tightly as in α_δ_, and a minor more open conformation, as seen in apo and α-bungarotoxin-bound structures (Supplementary Fig. 8c, d)^17,19,20^. Together the structural observations are consistent with different affinities for the two sites, observed with agonists like epibatidine^37,38^ and antagonists like snake toxins^39^ and *d*-tubocurarine^40^.

Binding of succinylcholine in the neurotransmitter sites coincides with the ion channel adopting a funnel-shaped conformation consistent with a desensitized state (Fig. 2c)^33,41^. This pore conformation is similar to that seen in the presence of etomidate. Moreover, both α-M4 C-termini are detached from the receptor’s coupling region in both structures (Fig. 2b, c, Supplementary Figs. 4 and 8e). The β, γ and δ subunits contain C-terminal extensions that interact with the receptor ECD and tether M4 in place, which may prevent their M4 helices from tilting away from the pore axis. Additionally, the movement of the α subunit M4 helices likely stems from those subunits undergoing the largest conformational changes between resting and desensitized states^19,20^.

These findings connect the activity of agonists like succinylcholine that stabilize a desensitized state to the activity of an allosteric antagonist like etomidate that binds to the same conformation and could thereby slow recovery to a resting, activatable state. We previously found that at high concentrations, *d*-tubocurarine was bound to one of the two etomidate sites^20^. However, *d*-tubocurarine exerts potent antagonist activity through binding at the classical neurotransmitter site, leading us to question whether occupancy of that allosteric site was an experimental artifact. The results with etomidate, in the context of the succinylcholine-bound receptor structure, help to support the α-M4 intrasubunit site as a relevant locus for negative allosteric modulation. Together, this pair of structures suggests that in clinical anesthesia, etomidate and succinylcholine should enhance each other’s muscle relaxant activities. We were curious to next contrast the mechanism of antagonism for a non-depolarizing blocker.

### Non-depolarizing blocker rocuronium stabilizes a resting-like state

**Fig. 3:**
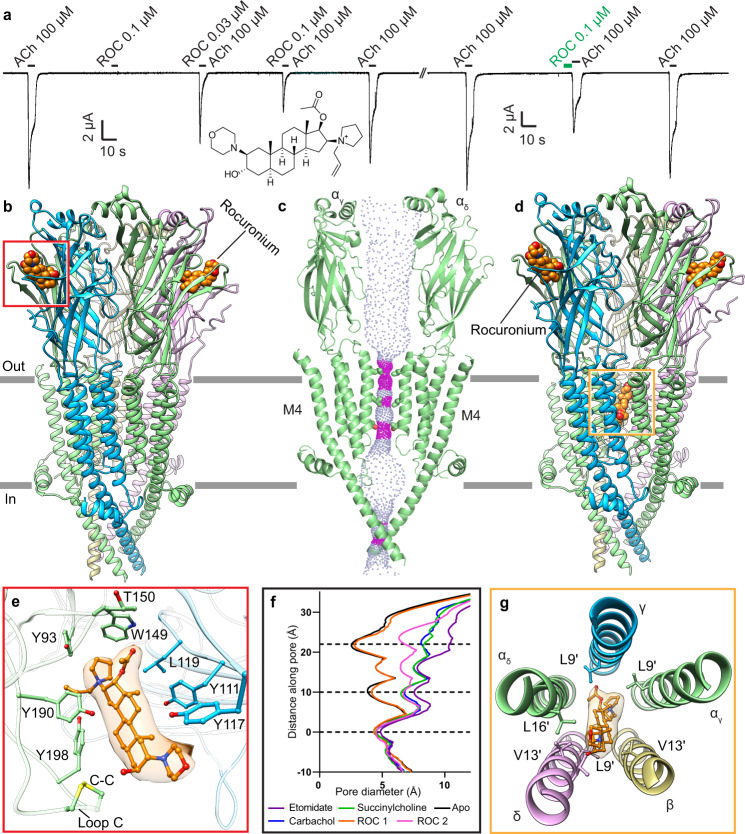
Rocuronium inhibition and binding mechanism. **a** TEVC recording illustrating inhibition of acetylcholine evoked currents by rocuronium (ROC, chemical structure). Second recording shows effect of rocuronium pre-application consistent with the antagonist being able to bind to the resting state. Similar responses were observed in *n* = 6 independent cells. **b** Side view of rocuronium-bound structure, with subunits colored as in Fig. 1 and rocuronium as orange spheres. **c** Side view of two α subunits showing permeation pathway of **b** as dots representing solvent-accessible surface colored by diameter. Constriction points are formed by T244, L255 and L258, which are shown as sticks. **e** Rocuronium at α/γ interface. **d** Side view of rocuronium pore-blocked structure, with subunits colored as in Fig. 1 and rocuronium as orange spheres. **e** Rocuronium at the α/δ interface. **f** Plot of distance along pore axis vs. pore diameter for structures in this study compared to the apo receptor structure (PDB: 7SMM), ROC 1 -rocuronium-bound resting-like state, ROC 2 rocuronium-bound pore-blocked state. **g** Rocuronium at pore site. Ligands and interacting residues are shown as sticks and corresponding densities of ligands are shown as semitransparent surfaces.

Rocuronium, introduced in 1994, belongs to a newer generation of aminosteroid neuromuscular blockers and has become the paralytic of choice for rapid sequence induction of anesthesia and intubation. It is a high-affinity competitive antagonist of the muscle-type nicotinic receptor^7^ and inhibits acetylcholine evoked whole cell currents both when pre-applied and co-applied with agonist (Fig. 3a). Rocuronium inhibits both muscle-type and neuronal nicotinic receptors. To explore binding determinants underlying specificity of this compound, we obtained an initial cryo-EM reconstruction of the muscle-type receptor bound to rocuronium at an overall resolution of 2.8 Å (Supplementary Fig. 2b, c). Further focused classification revealed two distinct states of the receptor, one with rocuronium bound in the pore and one without (Fig. 3, Supplementary Fig. 2). Both structures contained clear density for rocuronium in both classical neurotransmitter sites (Fig. 3b, d), with loop C propped open by the ligand (Fig. 3e) and the ECD in an expanded conformation characteristic of antagonist-bound and apo Cys-loop receptors^33^. Rocuronium primarily makes contacts with the principal (α_δ_ and α_γ_) subunits in both states. On the complementary side of the neurotransmitter binding pocket, its closest interaction is with γY111 (Fig. 3e). The decreased number of interactions on the complementary subunit compared to succinylcholine may explain why rocuronium is able to bind not only muscle-type but also neuronal type receptors^42^. The complementary subunits are less conserved in their ligand-binding residues, and accordingly a compound that primarily interacts with the principal subunits will be more promiscuous than one that relies heavily upon interactions with the complementary binding pocket.

In the conformation with rocuronium bound only in the neurotransmitter binding sites, the receptor adopts an overall conformation like the apo resting state. The extracellular domain is more expanded than seen in the agonist-bound structures, and the channel pore contains hydrophobic constrictions in its upper half (Fig. 3c, f). This extensive hydrophobic barrier was seen in previous apo and antagonist-bound structures (Supplementary Fig. 4) and would prevent ion permeation. Also indicative of a resting-like state are the positions of the α-M4 helices (Fig. 3b, Supplementary Fig. 4). In this rocuronium-bound structure, the upper position of the α-M4 helices retain map density sufficient for modeling and remain in close apposition to the coupling region, contrasting with desensitized states where one or more α-M4 helices are detached. Together, this first rocuronium complex shows how the non-depolarizing blocker prevents channel activation by competing with agonist, inhibits the conformational transition to an activated state, and stabilizes the pore in a resting-like nonconducting state.

In contrast, the second conformation of the rocuronium-bound receptor stabilizes a conformation intermediate between the resting and desensitized states. Density for pore blockers is typically poorly defined, as these tend to be low affinity sites, and the blocker often binds in multiple orientations. However, the density for rocuronium is remarkably clear, revealing only one possible orientation and set of interactions (Fig. 3d, g). Its binding deep in the pore causes the extracellular ends of all five M2 helices to dilate or tilt away from the channel axis (Fig. 3d, g and Supplementary Fig. 4). Breaking of the hydrophobic activation gate coincides with poorer local resolution in the extracellular ends of M2, which suggests that these helices become comparatively dynamic. The largest movement occurs in M2 of the α_γ_ subunit, which rocuronium packs against most closely (Fig. 3g). The relative widening of the upper half of the TMD pore, as seen in desensitized state structures, affects the conformation of the M4 helices as well. The M4 of the α_γ_ subunit detaches from the receptor in a manner similar to that in the carbachol-bound desensitized state, wherein only M4 of the α_δ_ subunit is detached (Supplementary Fig. 4)^20^. The tightest constriction in the pore is at the 2ʹ residue T244 (Fig. 3c, f), a position also associated with the desensitized state constriction (Supplementary Fig. 4). Thus, despite the extracellular domain adopting an antagonist-bound conformation, the transmembrane domain appears to be locked in a conformation reminiscent of agonist-bound desensitized states. Could pore block contribute meaningfully to the paralytic action of rocuronium? This site is likely too low in affinity to be relevant in a clinical setting, however the structure shows how a representative blocker binds and thereby stabilizes a unique receptor conformation where the ECD is in a resting-like state, while the TMD resembles a desensitized-like state.

## Discussion

**Fig. 4:**
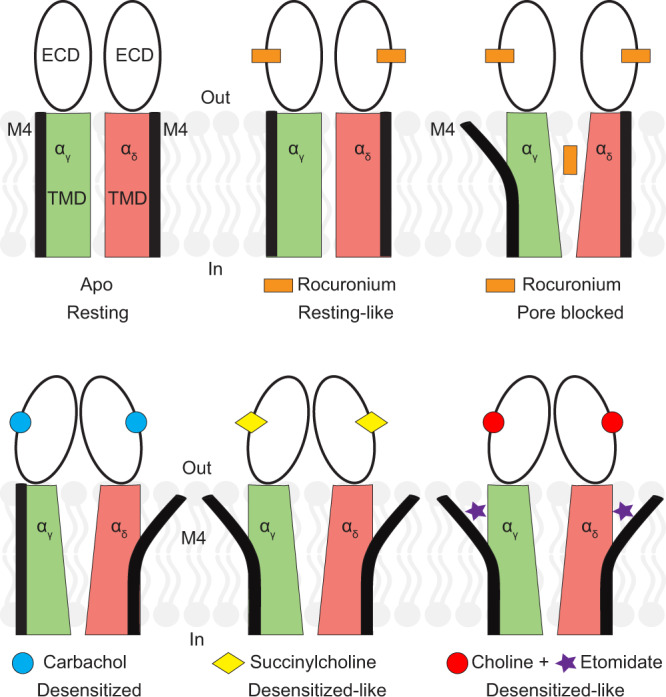
Agonist, blocker and anesthetic receptor sites and conformational changes. ECDs are shown as black hollow ellipses, α_γ_ subunit TMD is in green, the TMD of α_δ_ subunit is shown in red, embedded in lipid bilayer. M4 helices of both subunits are shown as thick black lines between the TMD and lipid bilayer.

Neuromuscular blockers paralyze skeletal muscle by antagonizing muscle nicotinic receptors; general anesthetics have also long been known to inhibit these excitatory ion channels. That these drug classes are frequently employed in combination makes it of interest to understand how they work and how they may either antagonize or enhance the other’s activity. To build a foundation for understanding drug interactions, we first mapped binding interactions for representative paralytics and an anesthetic. The atomic details of receptor interactions for the paralytic agents were largely unknown. We found that the depolarizing blocker succinylcholine binds in an extended conformation at both neurotransmitter sites and stabilizes a desensitized channel state. This finding is consistent with its activity as a low efficacy agonist and its stimulation of brief muscle contractions before producing flaccid paralysis. In contrast, rocuronium, a fast-acting non-depolarizing blocker, binds to the same two receptor sites, but interacts mainly with the principal (α) subunits, and stabilizes a resting channel state. Photoaffinity experiments with etomidate analogs have proposed a wealth of sites, but which ones matter, and the effect on receptor conformational states, have been largely shrouded in mystery. We found that the intravenous anesthetic etomidate binds in a largely unpredicted site, within the TMD bundle of α subunits, to stabilize a nonconducting desensitized-like state. These binding sites and correlated functional states are summarized in Fig. 4.

Emerging from these structures is an intrasubunit modulator site and mechanism for negative allosteric modulation. Previous structural studies found that in agonist bound or otherwise desensitized-like states, one or both of the α subunit M4 helices are detached from the body of the receptor (Supplementary Fig. 4). This conformational change may be a component of activation that persists during desensitization, or it may be triggered only during desensitization. In the presence of a high concentration of the desensitizing antagonist *d*-tubocurarine, this plant alkaloid can occupy one of the modulator sites, after saturating the higher affinity neurotransmitter site^20^. What remained unclear was whether this putative modulator site was relevant to a drug that exerted its principal activity through that site. The current study suggests that etomidate is an example of this drug class. The structures suggest that any ligand whose binding results in M4 detachment, and opening up of this modulator site, would enhance the inhibitory activity of etomidate; further, etomidate would enhance the tendency of an agonist to desensitize the receptor. Non-depolarizing blockers like rocuronium, in contrast, should allosterically inhibit etomidate binding; put another way, etomidate should increase the effective dose of rocuronium needed to compete off acetylcholine. Consequences in the clinic are likely unsubstantial for this case, as both ligands would still act to antagonize channel activity, and rocuronium is a much more potent antagonist than etomidate. Together, these structural studies provide a foundation for understanding the muscle relaxant effects of a representative intravenous anesthetic and how it may interact with commonly used neuromuscular blockers.

## Methods

### Receptor purification

Nicotinic acetylcholine receptor protein was purified from the electric organ of *T. californica* (EastCoast Bio) as previously described^15,17^. Briefly, 100 g of frozen tissue were thawed in 300 mL of buffer A (20 mM NaH_2_PO_4_, 400 mM NaCl, pH 7.4) supplemented with 150 mg NEM (N-ethylmaleimide, Sigma). Thawed tissue was homogenized and centrifuged at 3220 × *g* for 15 min. The supernatant was passed through cheesecloth and a protease inhibitor tablet (cOmplete mini, Sigma) was added to it with gentle stirring. Cell membranes were pelleted by centrifuging this supernatant at 105,000 × *g* for 30 min. The membrane pellet was collected and resuspended in buffer B (20 mM Tris, 80 mM NaCl, 1 mM EDTA, 20% sucrose, pH 11.0) and incubated on ice for 30 min. The resuspension was centrifuged at 105,000 × *g* for 30 min. The membrane pellet was washed twice with buffer C (20 mM NaH_2_PO_4,_ 80 mM NaCl, pH 7.4) and stored at −80 °C until further use. For protein purification, 2 g of the membrane pellet were thawed on ice. A homogenizer (Dounce) was used to resuspend the pellet in 50 mL of buffer C. Triton X-100 (1.5% v/v) and phenymethylsulfonyl fluoride (PMSF, 1 mM) were added to the sample, which was gently rocked for 1 h at 4 °C. The solubilized supernatant was collected by centrifuging the sample at 105,000 g for 30 min at 4 °C. 100 mL of buffer C were added to the supernatant. The affinity reagent ATM (2-[(4-aminobutanoyl)amino]-*N*,*N*,*N*-trimethylethanaminium) was coupled to NHS activated Sepharose resin (Cytiva) as reported previously^15,17^. Affinity resin (5 mL packed bed) was equilibrated with buffer C and mixed with diluted supernatant. The suspension was nutated for 1 h at 4 °C. Unbound sample was removed by washing the resin with buffer D (20 mM Tris, 80 mM NaCl, 1 mM EDTA, 1 mM *n*-dodecyl-β-D-maltoside, DDM, Anatrace, pH 7.4). For preparation of the receptor-succinylcholine sample, we used buffer E (20 mM Tris, 80 mM NaCl, 1 mM EDTA, 1 mM DDM, 50 mM βME, pH 7.4) containing 50 mM succinylcholine for elution. To prepare the rocuronium and etomidate samples, we used 1.2 M choline chloride (Sigma) as the eluting agent followed by the addition of respective ligands after elution (200 μM for both). Fluorescence size exclusion chromatography was used to analyze the quality of the elution fractions by monitoring tryptophan fluorescence. The eluted sample was concentrated to A280 = 7–8 using Amicon Ultra concentrators (Millipore) with a 100 kDa molecular weight cutoff.

### Reconstitution of protein into lipid nanodiscs

The sample was reconstituted into lipidic nanodiscs composed of soy polar lipids (Avanti Polar Lipids) and saposin. A molar ratio of receptor: saposin: soy polar lipids of 1:25:150 was used. Saposin A expression plasmid was kindly provided by Salipro Biotech^43^. For the rocuronium sample, cholesterol was added to the lipids in a ratio of 1:4. Briefly, the concentrated receptor was mixed with the lipid suspension and incubated at RT for 20 minutes, after which saposin was added. The reaction mix was incubated at RT for another 2 min. One hundred milligrams of Bio-Beads (SM-2 Bio-Rad) were washed once with methanol and three times with Milli-Q water and once with TBS (20 mM Tris, 80 mM NaCl, pH 7.4). Five hundred microliters of sample and washed biobeads were mixed and rotated overnight at 4 °C. Freshly washed Bio-Beads were replaced in the reaction tube and rotated for another 1–2 h. The reconstituted sample was aspirated using a 1 mL syringe and needle and ultracentrifuged at 98,600 × *g* for 30 min to remove precipitate or Bio-Beads. The sample was then passed through a Superose 6 10/300 GL Increase (Cytiva) column equilibrated with TBS to isolate the pentameric receptor species by removing empty nanodiscs and protein aggregates.

### Cryo-EM sample preparation

To obtain the succinylcholine-bound structure, 1 mM succinylcholine chloride (Sigma) was added to the TBS used in SEC. To obtain the rocuronium-bound structure, SEC elution fractions of apo receptor were supplemented with 200 μM rocuronium bromide (Tokyo Chemical Industry) both before and after concentrating. To obtain the etomidate and choline bound structure, 200 μM of etomidate (Tocris) was added only to SEC fractions. All samples were concentrated to an A_280_ of 7–8 and centrifuged at 98,600 × *g* to remove aggregates. Freshly prepared fluorinated Fos-Choline-8 (Anatrace) was added to the final sample at a concentration of 0.5 mM to induce random orientations of the protein molecules. 3 μL of sample were applied to Quantifoil R 1.2/1.3 (200 mesh, Copper) grids and plunge frozen in liquid ethane using a Vitrobot Mark IV (FEI).

### Cryo-EM data collection and processing

Cryo-EM grids were screened on the Talos Arctica at UT Southwestern Medical Center. Cryo-EM datasets were collected on 300 kV Titan Krios microscopes (FEI) at the PNCC (see Supplementary Table 1). Dose-fractionated micrographs were processed following a general RELION 3.1^44^ workflow. Structural biology software was compiled by SBgrid^45^. Pseudosymmetry did not pose problems during particle alignment, as described previously^20^. Movies were gain normalized, 2x Fourier binned, dose weighted, aligned and summed using MotionCor2^46^. Defocus values were estimated with GCTF^47^. CTF corrected particles were picked using crYOLO^48^. Good particles were selected using 2D classification. 2D classes with good structural features were used to generate a de novo initial model for 3D classification. Particles from 3D classes with strong intracellular domain (ICD) density were selected for further 3D refinement. The particles were polished, followed by a round of CTF refinement. A final 3D refinement and sharpening were performed. Focused classification after signal subtraction was used to resolve heterogeneity in the rocuronium sample. For the signal subtraction, a mask around the α_γ_ subunit was used.

### Model building, refinement and validation

The *d*-tubocurarine bound structure of the *Torpedo* receptor (PDB: 7SMR) was used as a starting model for succinylcholine and etomidate plus choline bound structures. The chemical structures of succinylcholine, etomidate, and choline were adapted from the PDB ligand repository. The apo structure of the *Torpedo* receptor (PDB ID: 7SMM) was used as a starting model for the rocuronium-bound structure. The geometry restraints for rocuronium were generated using the Grade Web Server (http://grade.globalphasing.org). Manual building and local refinement were performed in Coot^49^ and global real space refinement was performed in Phenix^50^. Stereochemistry and clashes were assessed using Phenix and the Molprobity^51^ server. Analysis of the ion pore was done using HOLE^52^. UCSF Chimera^53^, ChimeraX^54^ and Pymol (Schrodinger, LLC) were used to generate map and model figures.

### Electrophysiology

Open reading frames of the *Torpedo* α, β, γ and δ subunit-coding genes were subcloned into the pGH19 vector. The genes were kindly provided by Dr. Steven Sine from the Mayo Clinic. cRNAs were prepared from subcloned constructs using MEGAscript T7 Transcription kit (Thermo Fisher). *Xenopus laevis* ovary lobes were purchased from the National Xenopus Resource Center and maintained in Barth’s solution (in mM: 88 NaCl, 1 KCl, 2.4 NaHCO_3_, 0.82 MgSO_4_, 0.33 Ca(NO_3_)_2_, 0.68 CaCl_2_, 10 HEPES, pH 7.4) supplemented with 25 μg/mL ampicillin and 10 μg/mL gentamycin at 16 ^°^C. Ovarian lobes were cut into small pieces and washed with Barth’s solution. Cut lobes were digested with 1 mg/mL Type-I Collagenase (Gibco) in Barth’s solution supplemented with 1 mg/mL bovine serum albumin for 45–60 min. Digested ovarian lobes were shaken in 130 mM potassium hydrogen phosphate buffer, pH 6.5 (titrated by mixing monobasic and dibasic salts) for 15 min to facilitate release of oocytes from the lobes. Isolated oocytes were washed with ND96 solution (in mM: 96 NaCl, 2 KCl, 1 MgCl_2_, 1.8 CaCl_2_, and 5 HEPES pH 7.4) and analyzed under a microscope. Stage V-VI oocytes were isolated and stored in ND96 solution supplemented with 25 μg/mL ampicillin and 10 μg/mL gentamycin. Oocytes were micro-injected with 0.1–5 ng cRNA mixture of the subunits in the ratio 2:1:1:1 of α:β:γ:δ. Microinjections were performed in ND96 solution prepared without calcium. Approximately 24–72 h after injection, TEVC recordings were performed using an Axoclamp 900 A (Molecular Devices) amplifier and a Digidata 1550B (Molecular Devices) digitizer. Pipettes were polished to a resistance of 0.2–1.2 MΩ and filled with filtered 1 M KCl solution. ND96 was used as the bath solution and carrier of ligands. Data were analyzed using pClamp 10.7 (Molecular Devices).

### Reporting summary

Further information on research design is available in the Nature Portfolio Reporting Summary linked to this article.

## Supplementary information

Supplementary Information

Peer Review File

Description of Additional Supplementary Files

Supplementary Movie 1

Supplementary Movie 2

Supplementary Movie 3

Supplementary Movie 4

Supplementary Movie 5

Supplementary Movie 6

Supplementary Movie 7

Supplementary Movie 8

Supplementary Movie 9

Supplementary Movie 10

Reporting Summary

## Data Availability

The data that support this study are available from the corresponding authors upon request. Cryo-EM maps have been deposited in the Electron Microscopy Data Bank (EMDB) under accession codes EMD-28576 (rocuronium-bound resting-like state), EMD-28826 (rocuronium pore-blocked state), EMD-28892 (etomidate-bound desensitized-like state) and EMD-28893 (succinylcholine-bound desensitized-like state). Atomic model coordinates have been deposited in the Protein Data Bank (PDB) under accession code 8ESK (rocuronium-bound resting-like state), 8F2S (rocuronium pore-blocked state), 8F6Y (etomidate-bound desensitized-like state), and 8F6Z (succinylcholine-bound desensitized-like state). We have used the following published structures for comparison with our data: 6X3V, 7SMM, 7QKO, 6UWZ, 7Z14, 7SMR, 7QL6, 7QL5, 7SMS. Source data are provided with this paper.

## Source data

Source Data
