# Supplementary material for: Structural interplay of anesthetics and paralytics on muscle nicotinic receptors
Source: Nat Commun. 2023 Jun 1;14:3169. doi: 10.1038/s41467-023-38827-5 (PMC10235084; doi:10.1038/s41467-023-38827-5)

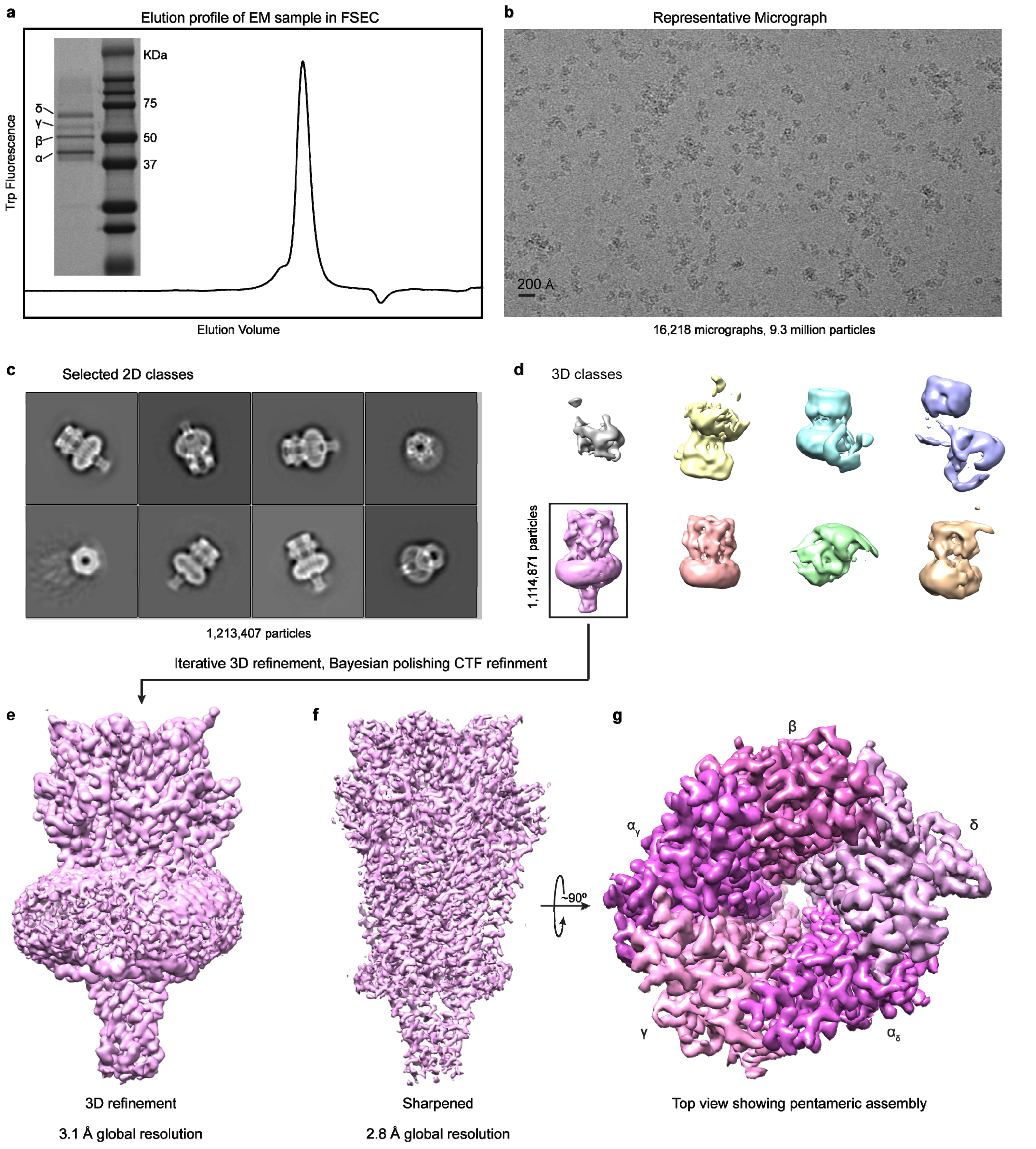

Supplementary Figure 1: Representative scheme of cryo-EM sample preparation and data processing for receptor-etomidate-choline complex. a, Analytical size-exclusion chromatography of EM sample showing pentamer peak; inset shows SDS-PAGE of the sample with four receptor subunits labelled. Results shown are representative of n ≥ 20 purifications. **b,** Micrograph showing distribution of particles on the grid; particles were picked using crYOLO. **c,** 2D classification of picked particles in RELION. Approximated 1.2 million particles were selected and used for 3D classification. **d,** Different 3 D classes obtained from RELION with selected 3D class shown in black box. **e,** Final 3D refinement map of etomidate-choline complex. **f**, **g,** Masked and sharpened map with global resolution at FSC=0.143.

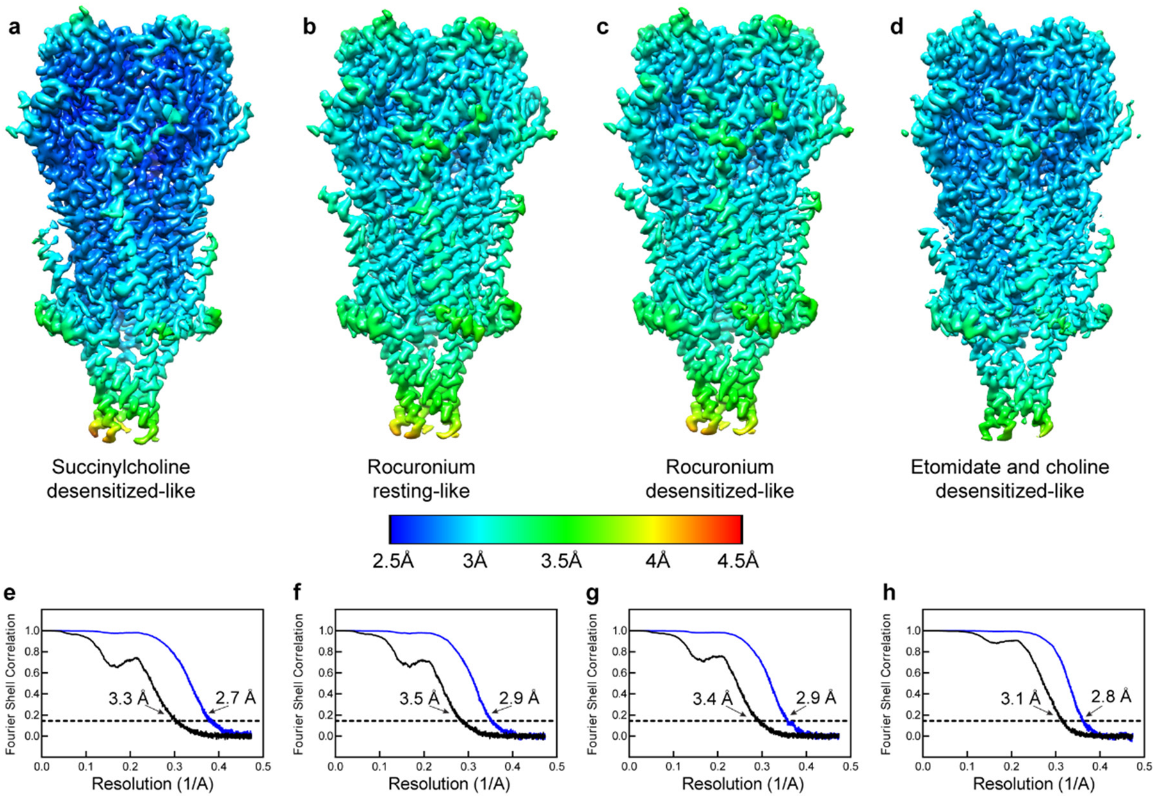

Supplementary Figure 2: Local and global resolution of cryo-EM maps. a-d, Local resolution maps of Torpedo muscle-type nicotinic receptor bound to listed ligands. **e-h**, Fourier shell correlation (FSC) plots for complexes listed above. Black and blue traces are for unmasked and masked maps, respectively. Resolution numbers are indicated at FSC=0.143.

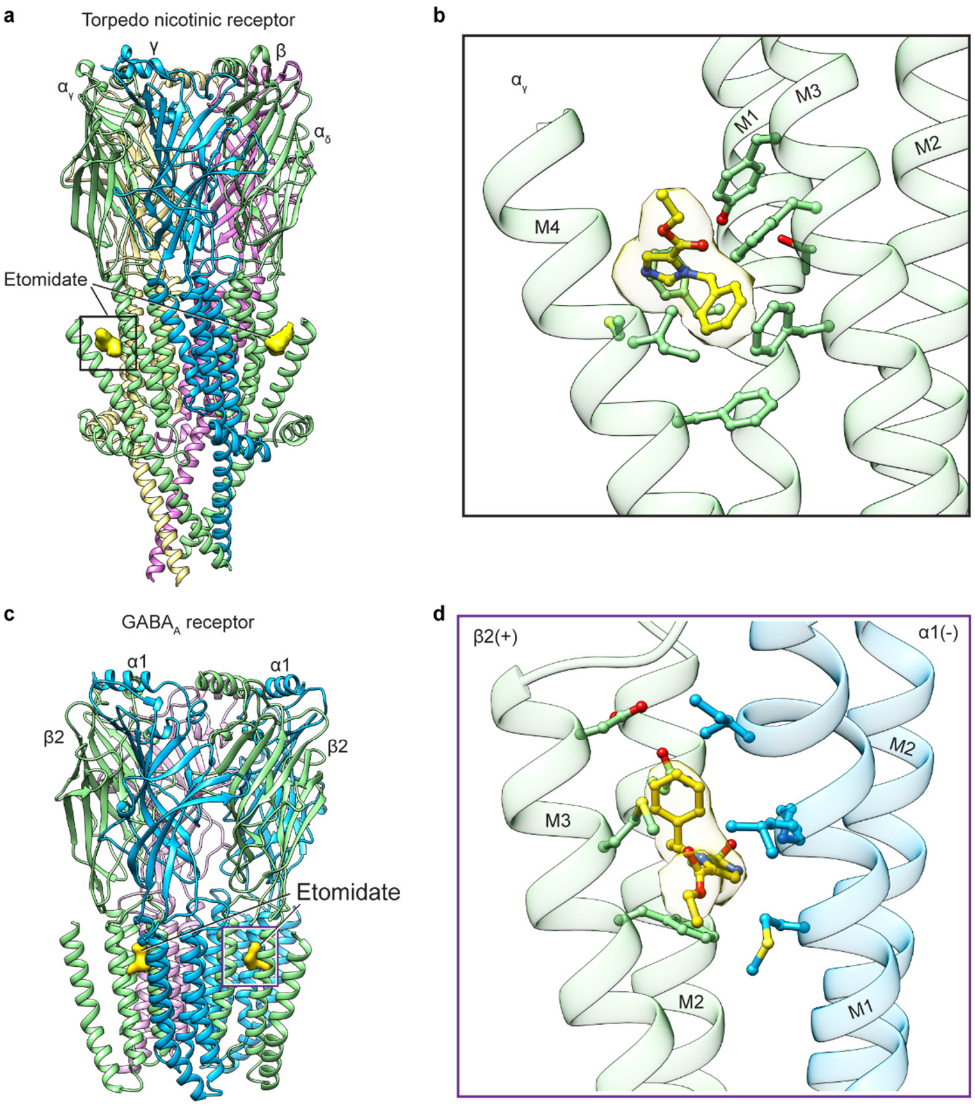

Supplementary Figure 3: Etomidate binding sites across Cys-loop receptors correlate with mechanism of action. **a,** *Torpedo* muscle type nicotinic acetylcholine receptor side view, colored by subunits, depicting etomidate densities as yellow surfaces in the intrasubunit site. **b,** Intrasubunit binding site of etomidate at the α_γ intrasubunit site. **c,** $GABA_A$ receptor bound to etomidate at the β2/α1 intersubunit site (PDB: 6X3V)[1]. **d,** Detail on binding site of etomidate at the β2/α1 interface.

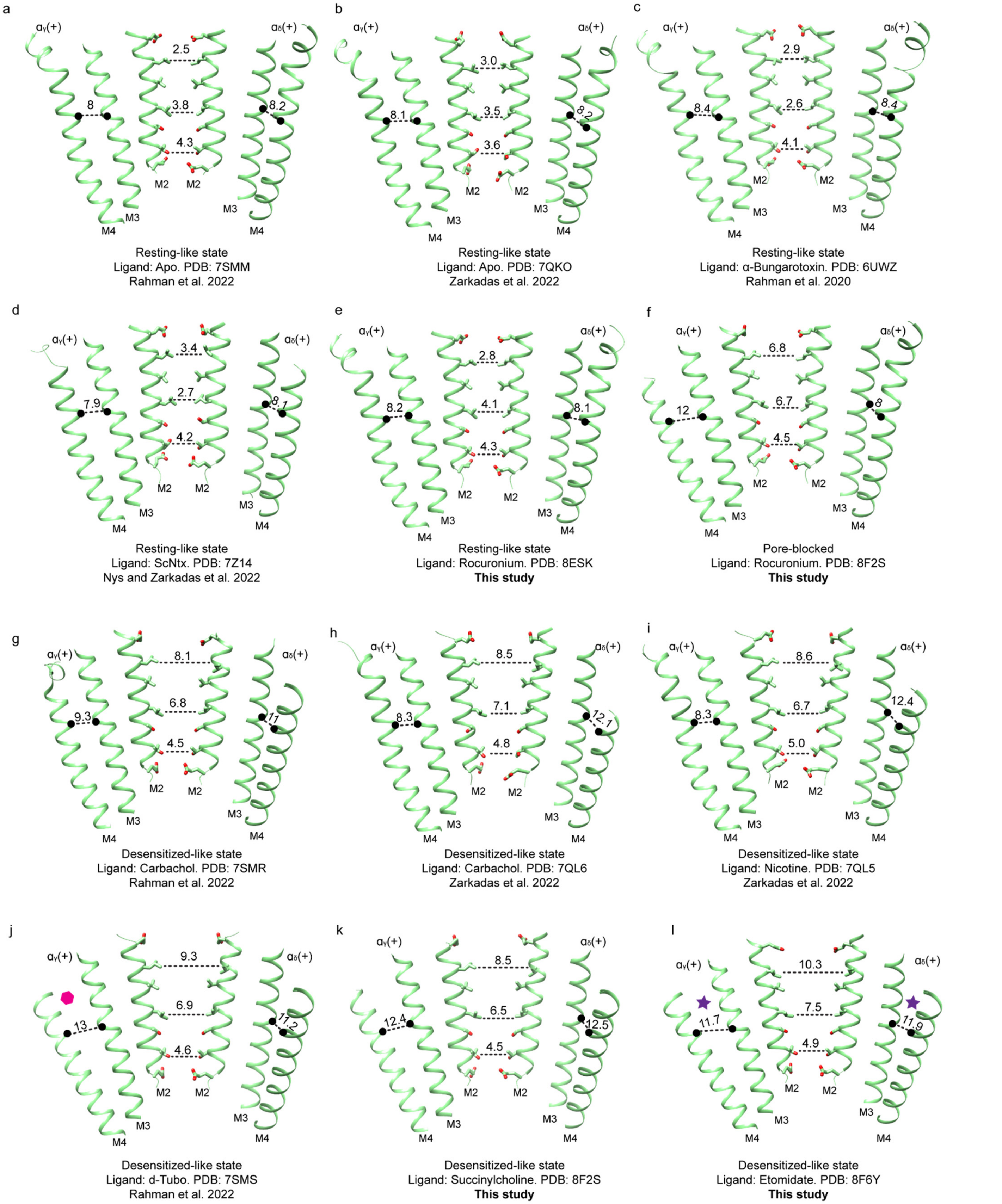
a
αγ(+)
αδ(+)
2.5
8
3.8
8.2
4.3
M2
M2
M3
M3
M4
M4
Resting-like state
Ligand: Apo. PDB: 7SMM
Rahman et al. 2022
b
αγ(+)
αδ(+)
3.0
8.1
3.5
8.2
3.6
M2
M2
M3
M3
M4
M4
Resting-like state
Ligand: Apo. PDB: 7QKO
Zarkadas et al. 2022
c
αγ(+)
αδ(+)
2.9
8.4
2.6
8.4
4.1
M2
M2
M3
M3
M4
M4
Resting-like state
Ligand: α-Bungarotoxin. PDB: 6UWZ
Rahman et al. 2020
d
αγ(+)
αδ(+)
3.4
2.7
7.9
8.1
4.2
M2
M2
M3
M3
M4
M4
Resting-like state
Ligand: ScNtx. PDB: 7Z14
Nys and Zarkadas et al. 2022
e
αγ(+)
αδ(+)
2.8
4.1
8.2
8.1
4.3
M2
M2
M3
M3
M4
M4
Resting-like state
Ligand: Rocuronium. PDB: 8ESK
This study
f
αγ(+)
αδ(+)
6.8
6.7
12
8
4.5
M2
M2
M3
M3
M4
M4
Pore-blocked
Ligand: Rocuronium. PDB: 8F2S
This study
g
αγ(+)
αδ(+)
8.1
6.8
9.3
11
4.5
M2
M2
M3
M3
M4
M4
Desensitized-like state
Ligand: Carbachol. PDB: 7SMR
Rahman et al. 2022
h
αγ(+)
αδ(+)
8.5
7.1
8.3
12.1
4.8
M2
M2
M3
M3
M4
M4
Desensitized-like state
Ligand: Carbachol. PDB: 7QL6
Zarkadas et al. 2022
i
αγ(+)
αδ(+)
8.6
6.7
8.3
12.4
5.0
M2
M2
M3
M3
M4
M4
Desensitized-like state
Ligand: Nicotine. PDB: 7QL5
Zarkadas et al. 2022
j
αγ(+)
αδ(+)
9.3
6.9
13
11.2
4.6
M2
M2
M3
M3
M4
M4
Desensitized-like state
Ligand: d-Tubo. PDB: 7SMS
Rahman et al. 2022
k
αγ(+)
αδ(+)
8.5
6.5
12.4
12.5
4.5
M2
M2
M3
M3
M4
M4
Desensitized-like state
Ligand: Succinylcholine. PDB: 8F2S
This study
l
αγ(+)
αδ(+)
10.3
7.5
11.7
11.9
4.9
M2
M2
M3
M3
M4
M4
Desensitized-like state
Ligand: Etomidate. PDB: 8F6Y
This study

Supplementary Figure 4: Transmembrane domain conformations. (a-l) M2, M3 and M4 helices of both α_γ and α_δ are shown for different structures. Pore-lining residues that form gates in various states of the ion channel, 2' (T244), 9' (L251) and 16' (L258), are shown as sticks. Pore diameters (Å) at different positions are indicated as measured by HOLE[2]. Also shown is the distance (Å) between the M3 and M4 helices measured between α-carbons of M4-C418 and M3-F284. *d*-Tubocurarine (*d*-Tubo) binding site is shown as a magenta hexagon and etomidate binding sites are shown as purple stars.

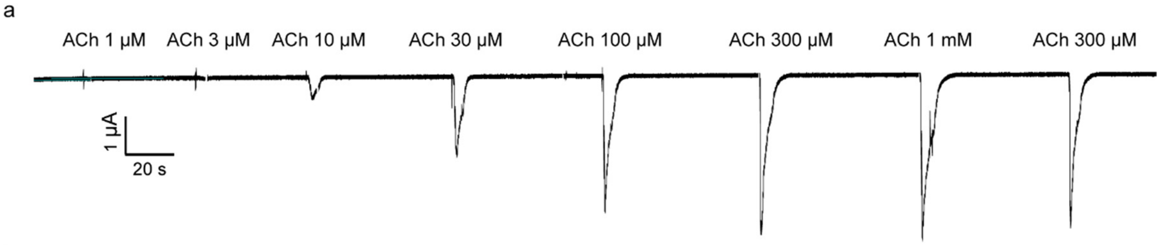

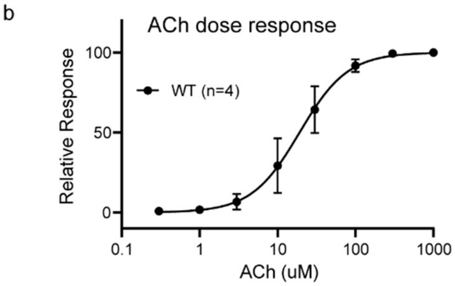

c

ACh dose response	EC_{50}(µM)	95% CI(µM)	EC_{80}(µM)	n
WT	19.18	15.92 to 23.09	54.94	4
G421A	51.06	45.47 to 57.34	140.9	4
I417A	27.75	23.17 to 33.17	71.18	5
C418A	36.02	32.30 to 40.20	93.38	4

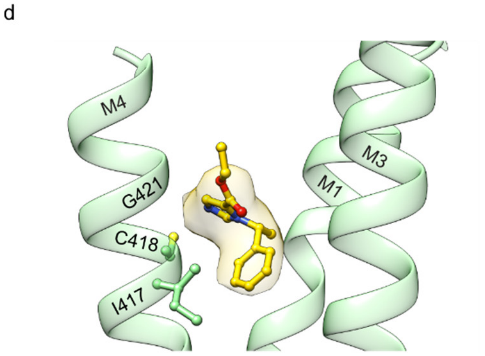

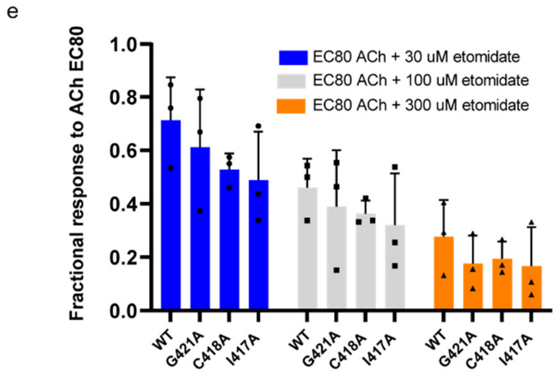

Supplementary Figure 5: Mutagenesis and two electrode voltage clamp tests of etomidate site residues. a, TEVC recording showing a representative trace from WT *Torpedo* receptor expressed in Xenopus oocytes and its response to increasing concentrations of acetylcholine. **b**, Dose response curve of WT receptor to acetylcholine from TEVC recordings. **c**, Acetylcholine EC_{50} and EC_{80} values for WT and mutant receptors calculated from dose response curves. **d**, Transmembrane M4 binding pocket of etomidate in the α subunit is shown with side chains of amino acids that were mutated for TEVC experiments. **e**, Mean fractional response of WT and mutant receptors to increasing concentration of etomidate compared with currents elicited by EC_{80} acetylcholine. n=3 for WT, G421A, I417A and C418A. Data points are shown as dots (●), squares (■) and triangles (▲), Mean is plotted, and standard error bars are shown. Fitting and calculations in **b, c**, and **e** were performed with GraphPad Prism.

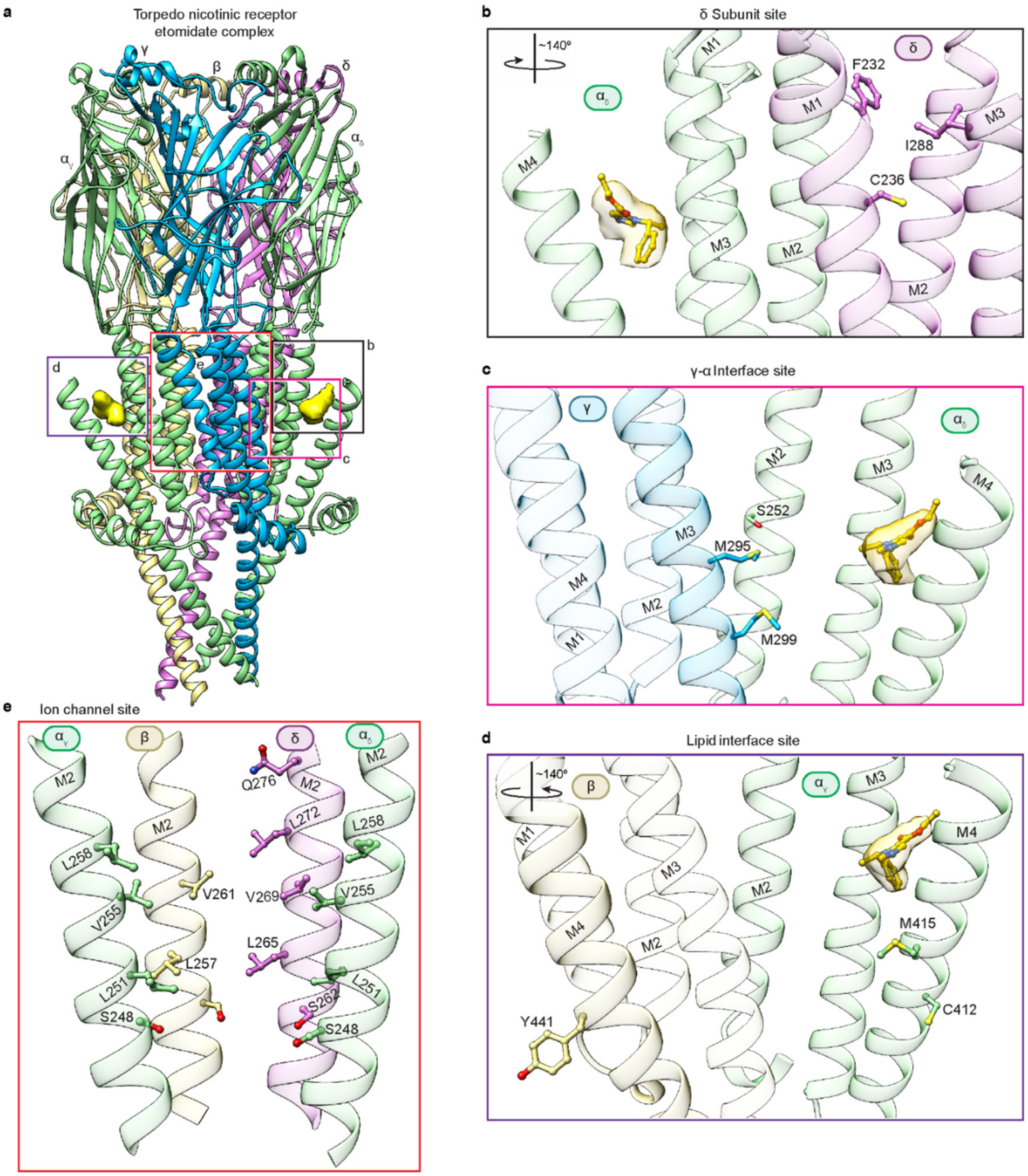

Supplementary Figure 6: Etomidate binding sites predicted by photolabeling experiments[3]. **a**, Side view of muscle-type nicotinic receptor from *Torpedo* showing four different types of etomidate binding sites reported using reactive etomidate derivatives. Classes of labeled sites are boxed in **a**, with details shown in **b-e**. Approximate rotations from perspective in **a** to views shown in **b** and **d** are indicated in upper-left corners of panels. Density for etomidate as observed in cryo-EM maps is shown as golden surfaces. **b,** Residues photolabelled by reactive etomidate in the TMD of the δ subunit. **c,** Photolabelled sites in the γ-α_δ TMD interface; M1 subunit is removed for clarity. **d,** Photolabelled sites in the α_γ and β subunits at the receptor-lipid interface. **e**, Residues labeled on M2 helices lining the TMD pore; γ subunit was removed for clarity. Model is colored as in main figures. EM experiments are consistent with a low affinity pore site indicated in **e** (Supplementary Fig. 6), but do not lend insight into the sites highlighted in **b-d**.

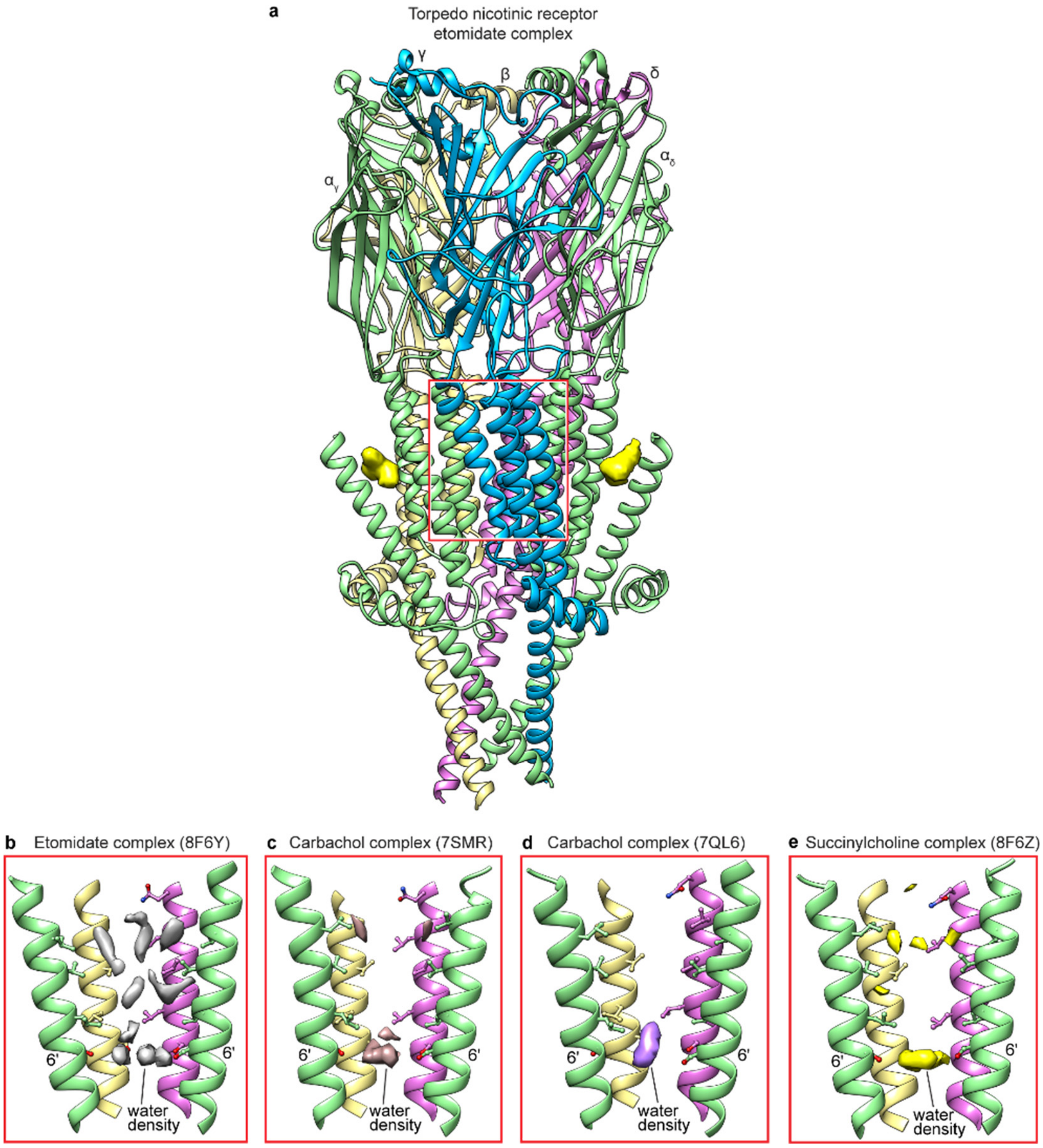

Supplementary Figure 7: Pore density is consistent with low affinity channel binding site for etomidate. a, Overall structure of *Torpedo* receptor – etomidate complex for reference; boxed region is expanded in lower panels. **b**-**d**, Detailed side views of pore-lining M2 helices from three different structures, with γ subunit removed for clarity. Experimental density map is shown in the pore at contour levels such that the density is ~equivalent for the water molecules seen nearby the 6' residues. Side chains are shown for residues indicated by photolabeling studies as potential determinants of etomidate inhibition. **b**, Structure of etomidate complex from the current study shows relatively strong but noisy density in the pore (threshold = 0.0152), nearby photolabelled residues, and not seen at similar relative contour levels in the compared structures. **c**, Carbachol-bound structure at 2.8 Å resolution[4] shows hints of density (threshold = 0.0174) near the extracellular end of the pore. **d**, Carbachol-bound structure at 3.1 Å resolution[5] shows no density (threshold = 0.158) in the central or upper pore region at this contour level. **e**, Succinylcholine-bound structure at 2.7 Å resolution shows noisy density (threshold = 0.0185) in the pore above the water ring.

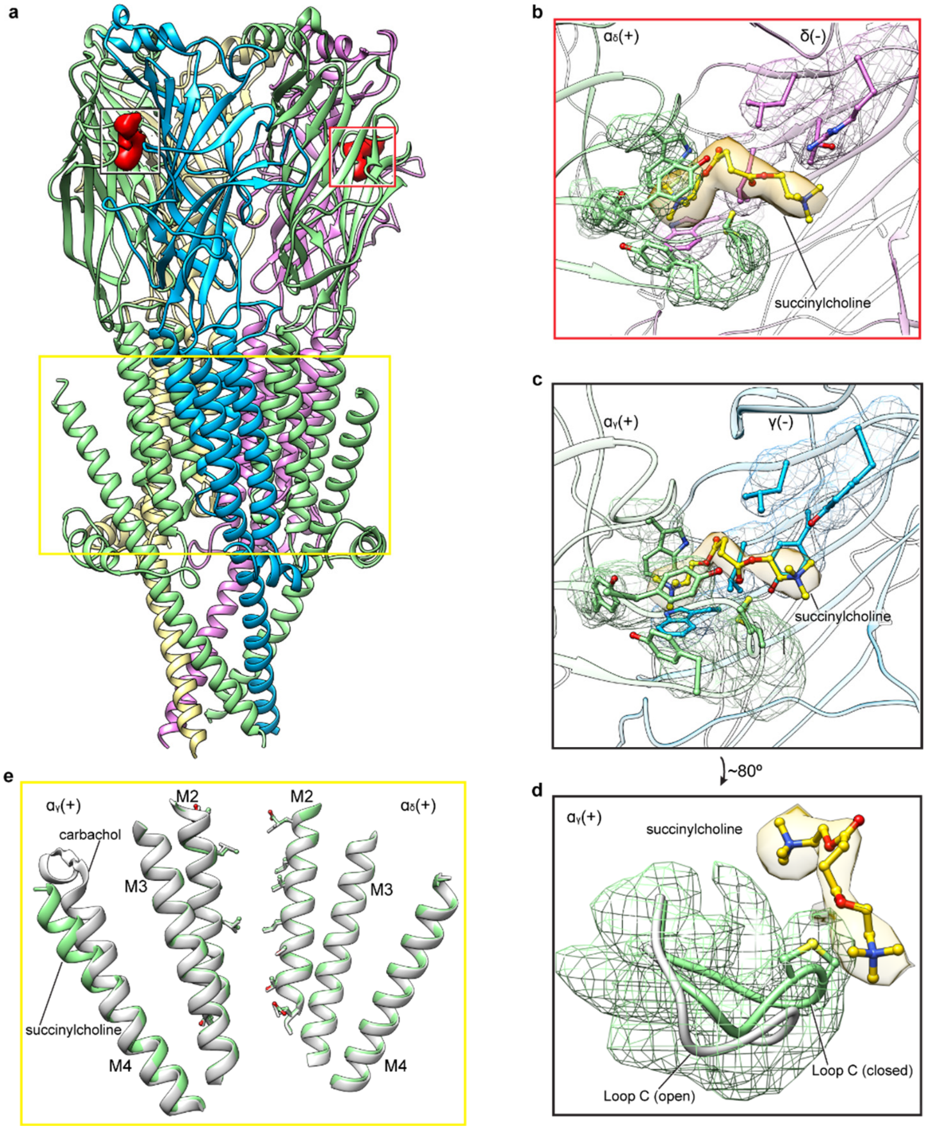

Supplementary Figure 8: Density quality and model fit of succinylcholine complex. **a**, Overall architecture. **b**, Side chain density quality in α_δ and δ subunit interface at the succinylcholine binding site. **c**, Side chain density quality in α_γ and γ subunit interface at the succinylcholine binding site. **d**, Loop C heterogeneity at α_γ-γ subunit interface. Grey is loop C from a resting state (apo, PBD 7SMM)[4]; green loop C is from succinylcholine complex. **e**, Compared to carbachol complex (gray), succinylcholine complex (green) has M4 of the α_γ subunit detached. Side chains are shown for M2 residues important in forming constrictions; these overlap in conformation between the two structures.

Supplementary Table 1. Cryo-EM data collection, refinement, and validation statistics

	Succinylcholine<br>PDB: 8F6Z<br>EMD-28893	Rocuronium (resting)<br>PDB: 8ESK<br>EMD-28576	Rocuronium (pore-blocked)<br>PDB: 8F2S<br>EMD-28826	Etomidate+choline<br>PDB: 8F6Y<br>EMD-28892
**Data collection and processing**				
**Magnification**	81,000	81,000	81,000	81,000
**Voltage (kV)**	300	300	300	300
**Electron exposure (e–/Å^2)**	50	50	50	50
**Defocus range (μm)**	0.8-2.0	0.5 - 2.5	0.5 - 2.5	0.8 – 2.0
**Pixel size (Å)**	1.0694	1.0694	1.0694	1.056
**Symmetry imposed**	C1	C1	C1	C1
**Initial particle images (no.)**	1,413,199	5,617,626	5,617,626	9,368,900
**Final particle images (no.)**	123,104	146,392	181,353	1,114,871
**Map resolution (Å)**<br>**FSC threshold = 0.143**	2.7	2.9	2.9	2.79
**Refinement**				
**Initial model used (PDB code)**	7SMR	7SMM	7SMM & 7SMR	7SMS
**Map sharpening *B* factor (Å^2)**	-25	-25	-25	-55
**Model composition**				
**Non-hydrogen atoms**	16,992	17,261	16,845	17,048
**Protein residues**	2015	2030	2023	2022
**Ligands**	2	2	3	4
***B* factors (Å^2)**				
**Protein**	49.98	72.37	93.18	66.99
**Ligand**	67.10	84.56	99.37	81.45
**R.m.s. deviations**				
**Bond lengths (Å)**	0.006	0.005	0.006	0.006
**Bond angles (°)**	0.586	0.578	0.566	0.572
**Validation**<br>**MolProbity score**<br>**Clashscore**<br>**Poor rotamers (%)**	1.63<br>8.66<br>0	1.23<br>1.91<br>0	1.40<br>3.16<br>0	1.42<br>5.21<br>0
**Ramachandran**<br>**Favored (%)**<br>**Allowed (%)**<br>**Disallowed (%)**	97.04<br>2.96<br>0	95.97<br>4.03<br>0	95.71<br>4.29<br>0	97.25<br>2.75<br>0

Supplementary References

1 Kim, J. J. *et al.* Shared structural mechanisms of general anaesthetics and benzodiazepines. *Nature* **585**, 303-308 (2020). https://doi.org:10.1038/s41586-020-2654-5

2 Smart, O. S., Neduvelil, J. G., Wang, X., Wallace, B. A. & Sansom, M. S. HOLE: a program for the analysis of the pore dimensions of ion channel structural models. *J Mol Graph* **14**, 354-360, 376 (1996). https://doi.org:10.1016/s0263-7855(97)00009-x

3 Hamouda, A. K., Stewart, D. S., Husain, S. S. & Cohen, J. B. Multiple transmembrane binding sites for p-trifluoromethyldiazirinyl-etomidate, a photoreactive Torpedo nicotinic acetylcholine receptor allosteric inhibitor. *J Biol Chem* **286**, 20466-20477 (2011). https://doi.org:10.1074/jbc.M111.219071

4 Rahman, M. M. *et al.* Structural mechanism of muscle nicotinic receptor desensitization and block by curare. *Nat Struct Mol Biol* (2022). https://doi.org:10.1038/s41594-022-00737-3

5 Zarkadas, E. *et al.* Conformational transitions and ligand-binding to a muscle-type nicotinic acetylcholine receptor. *Neuron* **110**, 1358-1370 e1355 (2022). https://doi.org:10.1016/j.neuron.2022.01.013

Original gel presented in supplementary figure 1

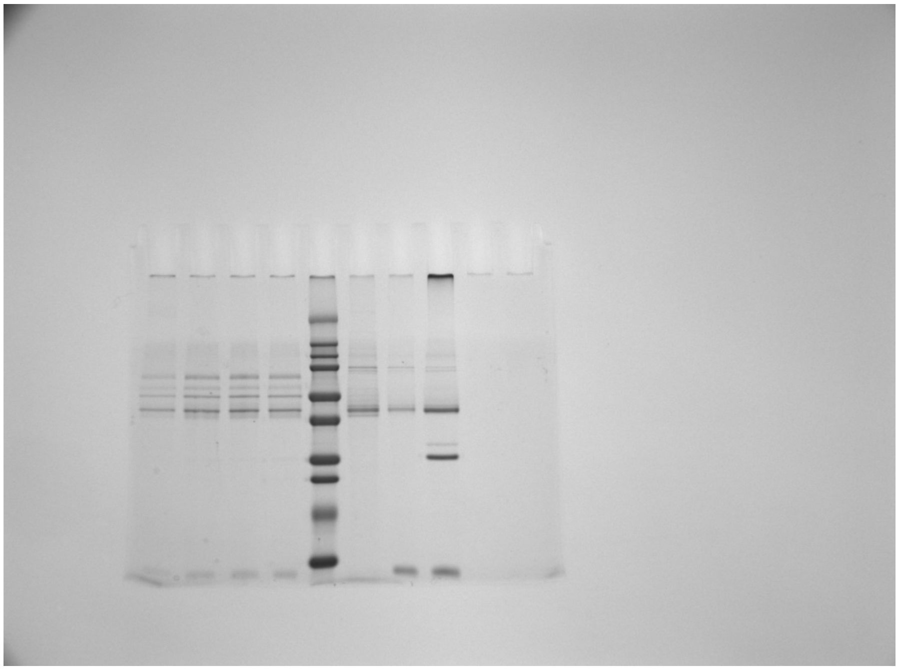

Supplement: Supplementary file 1 — Supplementary Information [file 41467_2023_38827_MOESM1_ESM.pdf]
