# Supplementary material for: Structural interplay of anesthetics and paralytics on muscle nicotinic receptors
Source: Nat Commun. 2023 Jun 1;14:3169. doi: 10.1038/s41467-023-38827-5 (PMC10235084; doi:10.1038/s41467-023-38827-5)
Supplement: Supplementary file 3 — Description of Additional Supplementary Files [file 41467_2023_38827_MOESM3_ESM.pdf]

Description of Additional Supplementary Files

File Name: Supplementary Movie 1
Description: Depicting density quality of etomidate and interacting amino acids at the αγ intrasubunit binding site

File Name: Supplementary Movie 2
Description: Depicting density quality of etomidate and interacting amino acids at the αδ intrasubunit binding site

File Name: Supplementary Movie 3
Description: Depicting density quality of choline and interacting amino acids at the αγ-γ subunit interface binding site

File Name: Supplementary Movie 4
Description: Depicting density quality of choline and interacting amino acids at the αδ-δ subunit interface binding site

File Name: Supplementary Movie 5
Description: Depicting density quality of rocuronium and interacting amino acids at the αγ-γ subunit interface binding site

File Name: Supplementary Movie 6
Description: Depicting density quality of rocuronium and interacting amino acids at the αδ-δ subunit interface binding site

File Name: Supplementary Movie 7
Description: Density quality of rocuronium and interacting amino acids in the ion pore

File Name: Supplementary Movie 8
Description: Density quality of succinylcholine and interacting amino acids at the αδ-δ subunit interface binding site

File Name: Supplementary Movie 9
Description: Density quality of succinylcholine and interacting amino acids at the αγ-γ subunit interface binding site

File Name: Supplementary Movie 10
Description: Density extending from M4-C418 close to bound etomidate at the αγ site
