# Supplementary material for: Structural interplay of anesthetics and paralytics on muscle nicotinic receptors
Source: Nat Commun. 2023 Jun 1;14:3169. doi: 10.1038/s41467-023-38827-5 (PMC10235084; doi:10.1038/s41467-023-38827-5)
Supplement: Supplementary file 14 — Reporting Summary [file 41467_2023_38827_MOESM14_ESM.pdf]

nature portfolio

Corresponding author(s): Ryan E. Hibbs

Last updated by author(s): April 28,2023

Reporting Summary

Nature Portfolio wishes to improve the reproducibility of the work that we publish. This form provides structure for consistency and transparency in reporting. For further information on Nature Portfolio policies, see our Editorial Policies and the Editorial Policy Checklist.

Statistics

For all statistical analyses, confirm that the following items are present in the figure legend, table legend, main text, or Methods section.

n/a	Confirmed	
☐	☒	The exact sample size (*n*) for each experimental group/condition, given as a discrete number and unit of measurement
☐	☒	A statement on whether measurements were taken from distinct samples or whether the same sample was measured repeatedly
☒	☐	The statistical test(s) used AND whether they are one- or two-sided<br>*Only common tests should be described solely by name; describe more complex techniques in the Methods section.*
☒	☐	A description of all covariates tested
☒	☐	A description of any assumptions or corrections, such as tests of normality and adjustment for multiple comparisons
☐	☒	A full description of the statistical parameters including central tendency (e.g. means) or other basic estimates (e.g. regression coefficient) AND variation (e.g. standard deviation) or associated estimates of uncertainty (e.g. confidence intervals)
☒	☐	For null hypothesis testing, the test statistic (e.g. *F*, *t*, *r*) with confidence intervals, effect sizes, degrees of freedom and *P* value noted<br>*Give P values as exact values whenever suitable.*
☒	☐	For Bayesian analysis, information on the choice of priors and Markov chain Monte Carlo settings
☒	☐	For hierarchical and complex designs, identification of the appropriate level for tests and full reporting of outcomes
☒	☐	Estimates of effect sizes (e.g. Cohen's *d*, Pearson's *r*), indicating how they were calculated

Our web collection on statistics for biologists contains articles on many of the points above.

Software and code

Policy information about availability of computer code

Data collection	SerialEM, Clampex, Axoclamp 900A Commander
Data analysis	RELION 3.1, crYOLO 1.7, MotionCor2 1.4, Coot 0.9.8, Chimera 1.16, ChimeraX 1.5, Phenix 1.19, ClampFit 11, GraphPad Prism 9, PyMol 2.5, HOLE2 2.2, Resmap 1.1, GCTF 1

For manuscripts utilizing custom algorithms or software that are central to the research but not yet described in published literature, software must be made available to editors and reviewers. We strongly encourage code deposition in a community repository (e.g. GitHub). See the Nature Portfolio guidelines for submitting code & software for further information.

Data

Policy information about availability of data

All manuscripts must include a data availability statement. This statement should provide the following information, where applicable:

- Accession codes, unique identifiers, or web links for publicly available datasets
- A description of any restrictions on data availability
- For clinical datasets or third party data, please ensure that the statement adheres to our policy

Cryo-EM maps and atomic model coordinates generated from this study have been deposited in the EMDB and RCSB respectively and will be released upon publication of the manuscript. Accession numbers are: PDB 8ESK [http://doi.org/10.2210/pdb8ESK/pdb] and EMD-28576 [https://www.ebi.ac.uk/pdbe/entry/emdb/EMD-28576] for the rocuronium-bound resting-like state, PDB 8F2S [http://doi.org/10.2210/pdb8F2S/pdb] and EMD-28826 [https://www.ebi.ac.uk/pdbe/entry/emdb/EMD-28826] for the rocuronium pore-blocked state, PDB 8F6Y [http://doi.org/10.2210/pdb8FY6/pdb] and EMD-28892 [https://www.ebi.ac.uk/pdbe/entry/emdb/EMD-28892] for the etomidate-bound desensitized-like state, PDB 8F6Z [http://doi.org/10.2210/pdb8F6Z/pdb] and EMD-28893 [https://www.ebi.ac.uk/pdbe/entry/emdb/EMD-28893] for the succinylcholine-bound desensitized-like state. Atomic models and EM maps are available for manuscript review purposes upon request. We have used the following published structures for comparison with our data: PDB ID 6X3V [http://doi.org/10.2210/pdb6X3V/pdb] etomidate bound to GABAA receptor, PDB ID: 7SMM [http://doi.org/10.2210/pdb7SMM/pdb] Apo Torpedo receptor, PDB ID 7QKO [http://doi.org/10.2210/pdb7QKO/pdb] Apo Torpedo receptor, PDB ID 6UWZ [http://doi.org/10.2210/pdb6UWZ/pdb] α-bungarotoxin bound resting like Torpedo receptor, PDB ID 7Z14 [http://doi.org/10.2210/pdb7Z14/pdb] short chain neurotoxin bound resting like Torpedo receptor , PDB ID7SMR [http:// doi.org/10.2210/pdb7SMR/pdb] carbachol bound desensitized Torpedo receptor, PDB ID 7QL6 [http://doi.org/10.2210/pdb7QL6/pdb] carbachol bound desensitized Torpedo receptor, PDB ID 7QL5 [http://doi.org/10.2210/pdb7QL5/pdb] nicotine bound Torpedo receptor, PDB ID 7SMS [http://doi.org/10.2210/pdb7SMS/pdb d-tubo bound Torpedo receptor.The source data underlying supplementary figure 5b and 5e are provided as source data file.

Human research participants

Policy information about studies involving human research participants and Sex and Gender in Research.

Reporting on sex and gender	N/A
Population characteristics	N/A
Recruitment	N/A
Ethics oversight	N/A

Note that full information on the approval of the study protocol must also be provided in the manuscript.

Field-specific reporting

Please select the one below that is the best fit for your research. If you are not sure, read the appropriate sections before making your selection.

☒ Life sciences ☐ Behavioural & social sciences ☐ Ecological, evolutionary & environmental sciences

For a reference copy of the document with all sections, see nature.com/documents/nr-reporting-summary-flat.pdf

Life sciences study design

All studies must disclose on these points even when the disclosure is negative.

Sample size	Sample sizes were determined through preliminary experiments and authors' previous experiences
Data exclusions	RELION-based 2D and 3D classifications were used to remove junk particles from single particle cryo-EM datasets.
Replication	Experiments were replicated successfully as described in the legends
Randomization	EM particle sets were randomly split during processing and resolution estimation.
Blinding	The studies were not blinded.

Reporting for specific materials, systems and methods

We require information from authors about some types of materials, experimental systems and methods used in many studies. Here, indicate whether each material, system or method listed is relevant to your study. If you are not sure if a list item applies to your research, read the appropriate section before selecting a response.

Materials & experimental systems

n/a	Involved in the study
☒	☐ Antibodies
☒	☐ Eukaryotic cell lines
☒	☐ Palaeontology and archaeology
☒	☐ Animals and other organisms
☒	☐ Clinical data
☒	☐ Dual use research of concern

Methods

n/a	Involved in the study
☒	☐ ChIP-seq
☒	☐ Flow cytometry
☒	☐ MRI-based neuroimaging
